# The learning curve of the MS-TRAM/DIEP breast reconstruction by dual-trained breast surgeons

**DOI:** 10.1186/s12893-024-02344-z

**Published:** 2024-02-14

**Authors:** Shunrong Li, Songliang Zhang, Xiaolan Zhang, Jingwen Yan, Shuai Wang, Luyuan Tan, Nanyan Rao, Kai Chen, Liling Zhu

**Affiliations:** 1 Guangdong Provincial Key Laboratory of Malignant Tumor Epigenetics and Gene Regulation, Sun Yat-Sen Memorial Hospital, Sun Yat-Sen University, Guangzhou, 510120 China; 2 Breast Tumor Center, Sun Yat-sen Breast Tumor Hospital, Sun Yat-sen Memorial Hospital, Sun Yat-sen University, Guangzhou, Guangdong 510120 China; 3 Guangzhou Concord Cancer Center, Guangzhou, Guangdong 510100 China; 4 Department of Thyroid and Breast Surgery, The Second Hospital of Anhui Medical University, Hefei, China; 5 Artificial Intelligence Lab, Sun Yat-sen Memorial Hospital, Sun Yat-sen University, Guangzhou, Guangdong 510120 China

**Keywords:** Dual-trained breast surgeon, Breast reconstruction, MS-TRAM/DIEP, Autologous free flap, Learning curve

## Abstract

**Background:**

Breast cancer surgeries involving MS-TRAM/DIEP breast reconstruction has traditionally been collaborative efforts between breast surgeons and plastic surgeons. However, in our institution, this procedure is performed by dual-trained breast surgeons who are proficient in both breast surgery and MS-TRAM/DIEP breast reconstruction. This study aims to provide insights into the learning curve associated with this surgical approach.

**Materials and methods:**

We included eligible breast cancer patients who underwent MS-TRAM/DIEP breast reconstruction by dual-trained breast surgeons between 2015 and 2020 at our institution. We present the learning curve of this surgical approach, with a focus on determining factors affecting flap harvesting time, surgery time, and ischemic time. Additionally, we assessed the surgical complication rates.

**Results:**

A total of 147 eligible patients were enrolled in this study. Notably, after 30 cases, a statistically significant reduction of 1.7 h in surgery time and 21 min in ischemic time was achieved, signifying the attainment of a plateau in the learning curve. And the major and minor complications were comparable between the early and after 30 cases.

**Conclusion:**

This study explores the learning curve and feasibility experienced by dual-trained breast surgeons in performing MS-TRAM/DIEP breast reconstruction.

**Trial registration:**

NCT05560633.

**Supplementary Information:**

The online version contains supplementary material available at 10.1186/s12893-024-02344-z.

## Background

For patients with breast cancer who received mastectomy, autologous MS-TRAM/DIEP breast reconstruction using muscle-sparing free transverse rectus abdominal muscle (MS-TRAM) flap or deep inferior epigastric perforator (DIEP) flap has become more and more popular. In the traditional MS-TRAM/DIEP breast reconstruction that was performed after a mastectomy or a skin-sparing mastectomy, an oval/round skin paddle on the flap was preserved for post-operative direct visual monitoring (DVM). However, the sacrifice of the nipple-areolar-complex (NAC) significantly compromised the cosmetic outcome. Since the oncological safety of nipple-sparing mastectomy (NSM) in eligible patients has been confirmed in our previous study [1] as well as others [2–4], incorporation of NSM into MS-TRAM/DIEP breast reconstruction has been attempted [5–7].

However, the MS-TRAM/DIEP surgery is a kind of plastic surgery which is a very dedicated microsurgery technique with a wide spectrum of difficulties and complicated procedures [8]. Traditional MS-TRAM/DIEP breast reconstruction is a combination surgery between the breast surgeon and the plastic surgeon. In order to improve the cosmetic outcome, dual-trained breast surgeon concept come up for discussion [9]. How is the learning curve of the dual-trained breast surgeon had not been reported. At our center, there were dual-trained breast surgeons starting to perform MS-TRAM/DIEP breast reconstruction since 2015. The learning curve of this surgical approach, as well as the determining factors for surgical outcomes, and the oncological outcomes and the complication rates were reported.

## Materials and methods

### Study population

This single-center, retrospective study (NCT05560633) included the following patients: (1) Female breast cancer patients with age > 18 years; (2) Received autologous MS-TRAM/DIEP breast reconstruction (including muscle-sparing free TRAM or DIEP) in Sun Yat-sen Memorial Hospital between May 1st,2015 and December 31st,2020. Patients who received abdominal autologous pedicled-flap breast reconstruction were excluded. We collected the baseline demographic characteristics, such as BMI, Neoadjuvant Chemotherapy, ER, PR,HER2,cTNM-stage, pTNM-stage, parity, parity times, purpose of the surgery (Reconstruction or Repair), timing of the breast reconstruction surgery (Delayed, Immediate, Immediate-delayed ) and types of the breast cancer surgery (Modified Radical Mastectomy, Nipple Sparing Mastectomy and Skin Sparing Mastectomy), types of the abdominal free flap (DIEP, ms-TRAM), types of skin leaving on the flap (Buried Flap and DIEP with Skin Paddle) and Microscope (10X microscope, 3.5X loupes ).

Dual-trained breast surgeons were defined as surgeons who have received training in both breast surgery and plastic surgery. These surgeons are proficient in performing both mastectomy and flap harvesting procedures. This research has been approved by the ethical committees of Sun Yat-sen Memorial Hospital. (Number: SYSEC-KY-KS-2021-144).

### Study endpoint

The learning curve is measured as the variation of the flap harvest time, ischemic time and surgery time. Major complications included the take-back surgery and the total flap failure. Minor complications include the flap complications (e.g. liposclerosis, and flap volume decrease), the breast skin envelope complications (e.g. skin pocket necrosis) and the abdominal complication (e.g. complications of the abdominal incision and/or incisional hernia).

### Data analysis

We used median and interquartile range (IQR) for descriptive analysis of continuous variables. Mann-Whitney U test and Fisher exact test were used to compare the continuous and categorical variables between groups, respectively. We used univariate and multivariate linear regression to analyze the determinants of surgery time and ischemic time. Statistical analyses were performed with R statistical software and statistical significance was set at *P* < 0.05.

## Results

### Summary of demographic data

Among the 147 eligible patients for this study, the median (IQR) BMI was 23.1 (21.1–25.8) kg/m^2^. There were 18 and 129 cases in the buried-flap cohort and skin-paddle cohort, respectively. Among the eligible patients, DIEP accounted for 78.9% (116 cases), MS-TRAM accounted for 17.6% (26 cases), and there were 5 cases for which data could not be obtained. Most of the cases of our center were immediate (90.5%) breast reconstruction. (Table 1).

**Table 1:** Base line of patients enrolled in this study

	level	Overall n (%)
Patients		147 (100.0)
Neoadjuvant Chemotherapy (%)	No	76 (51.7)
	Yes	62 (42.2)
	NA	9 (6.1)
ER (%)	Negative	42 (28.6)
	Positive	98 (66.7)
	Unknown	7 (4.8)
PR(%)	Negative	56 (38.1)
	Positive	85 (57.8)
	Unknown	6 (4.1)
Her2 (%)	Negative	99 (67.3)
	Positive	36 (24.5)
	Unknown	12 (8.2)
cTNM-stage (%)	0	1 (0.7)
	I	8 (5.4)
	II	58 (39.5)
	III	32 (21.8)
	IV	38 (25.9)
	Unknown	10 (6.8)
pTNM-stage (%)	0	5 (3.4)
	I	21 (14.3)
	II	35 (23.8)
	III	35 (23.8)
	IV	39 (26.5)
	Unknown	12 (8.2)
Parity (%)	No	10 (6.8)
	Yes	128 (87.1)
	NA	9 (6.1)
Parity_times (%)	More than once	43 (29.3)
	None	10 (6.8)
	Once	56 (38.1)
	NA	9 (6.1)
Type of surgery (Purpose) (%)	Reconstruction	106 (72.1)
	Repair	41 (27.9)
Timing of breast reconstruction surgery (%)	Delayed	12 (8.2)
	Immediate	133 (90.5)
	Immediate-delayed	2 (1.4)
Type of the Breast Cancer Surgery (%)	(Modified) Radical Mastectomy	67 (45.6)
	Nipple Sparing Mastectomy	25 (17.0)
	Skin Sparing Mastectomy	55 (37.4)
Type of the Abdominal Free Flap (%)	DIEP	116(78.9)
	ms-TRAM	26(17.7)
	NA	5(3.4)
Type of Skin Leaving on the Flap (%)	Buried Flap	18 (12.2)
	DIEP with Skin Paddle	129(85.7)
Microscope (%)	10X microscope	61(41.5)
	3.5X loupes	86(58.5)

Abbreviation: BMI, Body-mass index; IQR, inter-quartile range; ER, estrogen receptor; PR, progesterone receptor; HER2, human epidermal growth factor receptor 2; DIEP, deep inferior epigastric perforator; ms-TRAM, muscle-sparing free transverse rectus abdominal muscle; NA, Not Available

### Learning curve

**Fig. 1:**
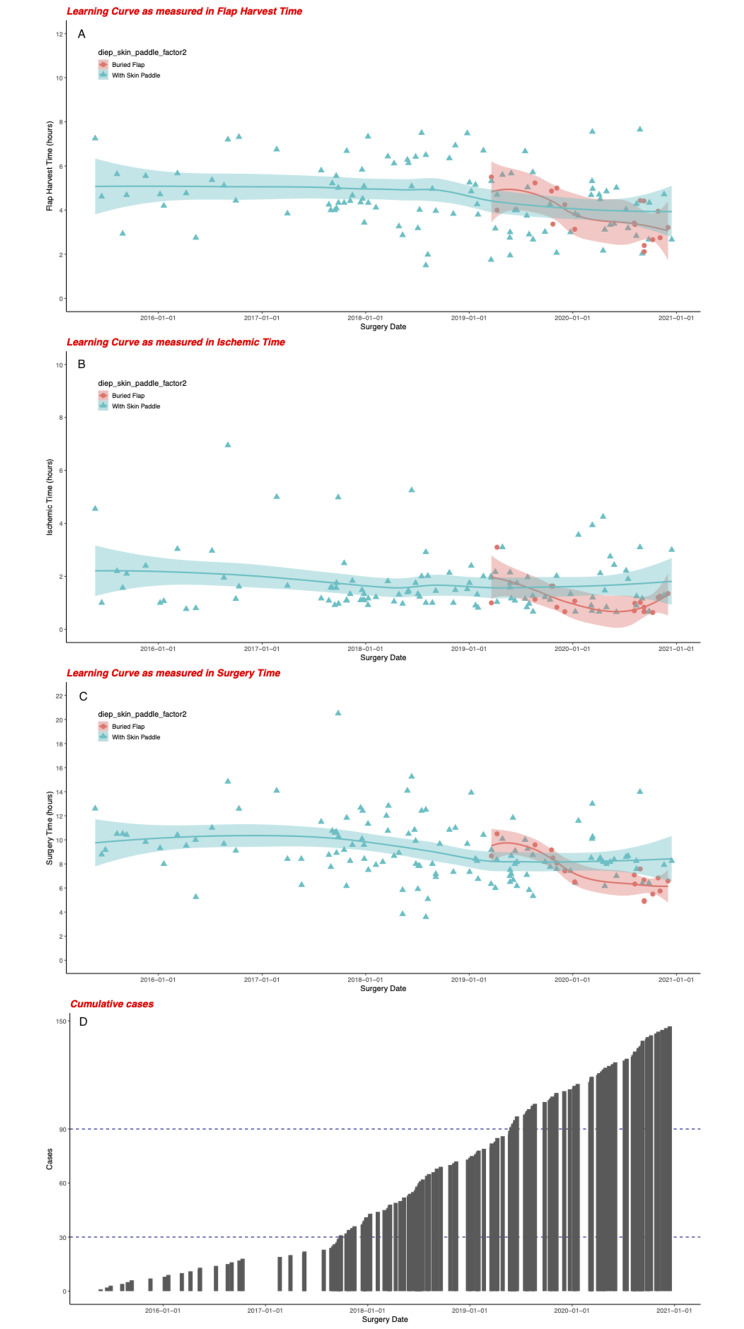
The learning curve analysis of ms-TRAM/DIEP breast reconstruction in our institution using the flap harvest time; (**A**) ischemic time (**B**) and surgery time (**C**) measurement of the performance. (D) Histogram to show the cumulative cases of ms-TRAM/DIEP breast reconstruction

**Fig. 2:**
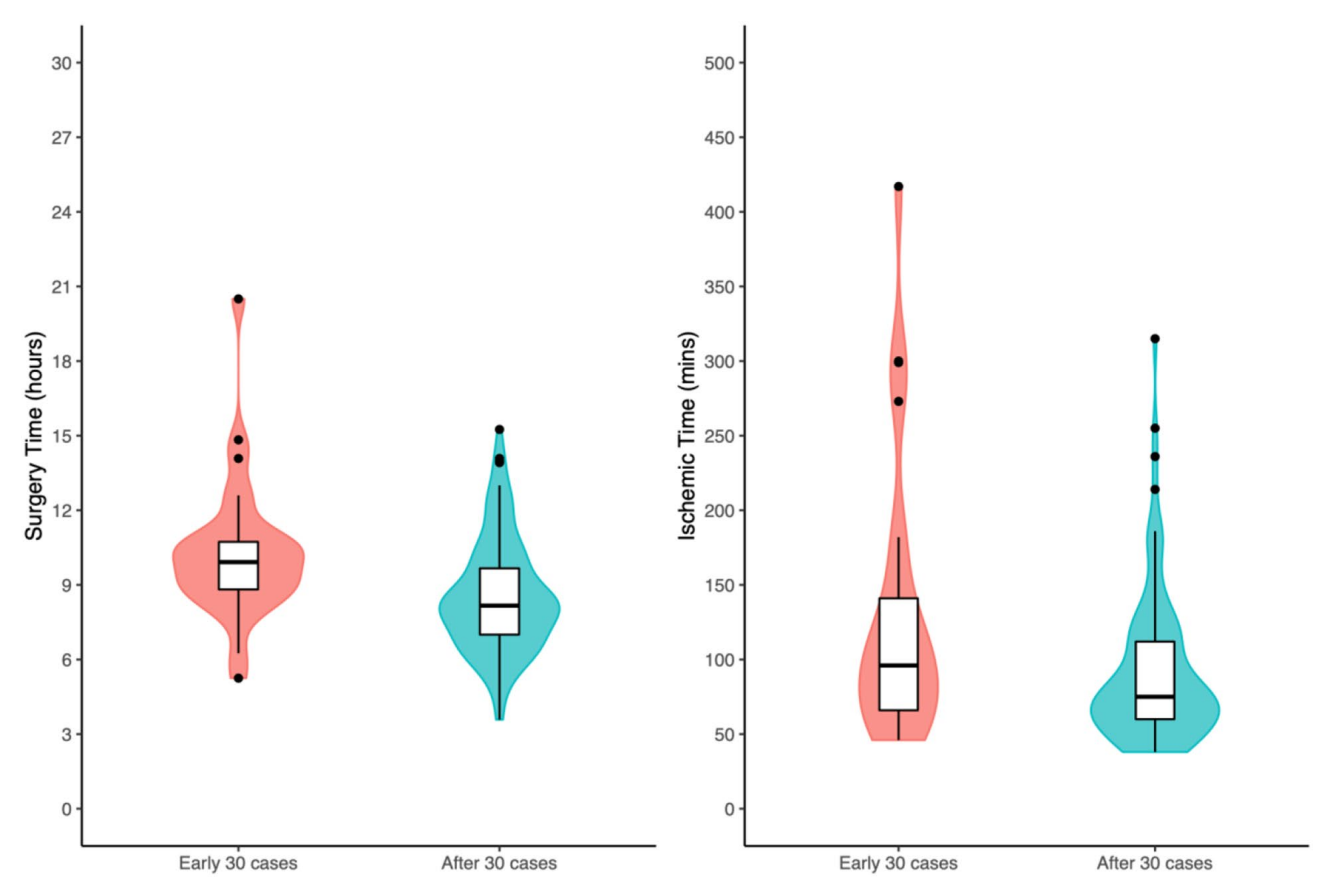
The surgery time and ischemic time were significantly shorter after the accumulation of 30 cases of ms-TRAM/DIEP experience

**Fig. 3:**
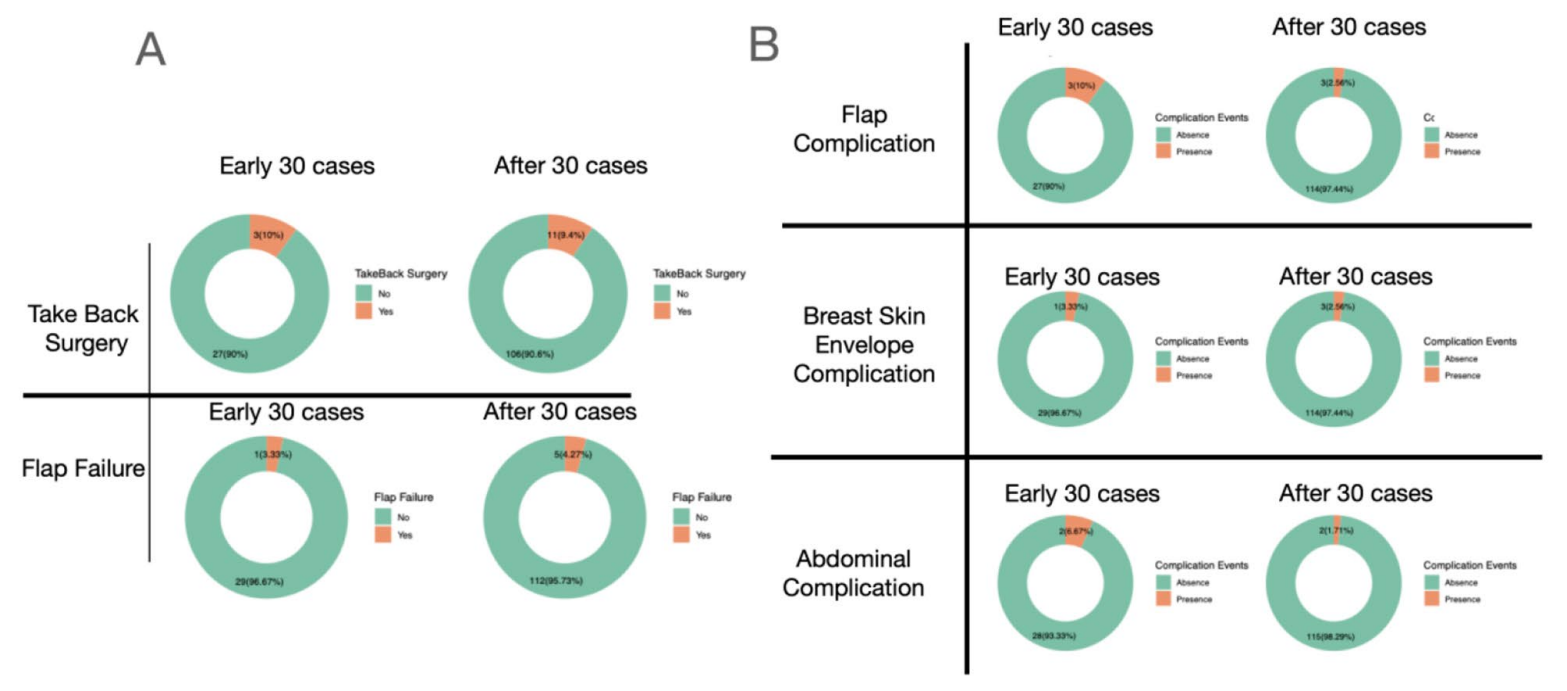
(**A**) The major complication rate of the Early 30 cases and After 30 cases. (**B**) The Minor complication rate of the of the Early 30 cases and After 30 cases

The learning curves were defined by the flap harvesting time, surgery time and ischemic time and the cumulative cases of MS-TRAM/DIEP were counted chronologically (Fig. 1). The first 30 cases [10] were conducted within 2 years (15 cases/year) and the median (IQR) surgery time and ischemic time were 9.9 (8.8–10.7) hours and 96 (66–141) minutes, respectively. Since the 31st case, the median (IQR) surgery time and ischemic time were 8.2 (7.0-9.7) hours and 75 (66–122) minutes, respectively. The difference of the surgery time and ischemic time between the two periods were statistically significant (Fig. 2). Regarding the complication rates, there were no significant differences between the major complication rates in between the first 30 cases period, and the after 30 cases period (Fig. 3A and B).

Next, we analysis of surgery time and ischemic time determinants. We performed univariate and multivariate analysis to identify the determinants of the operative time and ischemic time. As shown in Table 2A and 2B, we observed that higher pN stages and increasing age were significantly associated with longer operative times (*P* < 0.01) and ischemic time (*P* = 0.02), respectively. It should be noted that the 3.5X loupes (vs. 10x microscope) was significant associated with shorter surgery and ischemic times (*P* < 0.01).

**Table 2A:** A The uni- and multi- variate analysis of the surgery time determinants

	Univariate		Multivariate	
	estimate	*p*. value	estimate	*p*. value
**Age**	0.018	0.412		
**BMI**	0.078	0.197		
**Neoadjuvant Chemotherapy**				
No	0			
Yes	-0.298	0.482		
Unknown	0.035	0.968		
**pT Stage**				
pT0/T1/T2	0			
pT3/T4	-0.187	0.689		
pTx	-0.162	0.782		
**pN Stage**				
N0	0		0	
N1-N3	1.226	0.005	1.06	0.01
Unknown	0.349	0.621	0.701	0.31
**Estrogen Receptor**				
Negative	0			
Positive	0.532	0.291		
Unknown	1.062	0.305		
**Progesterone Receptor**				
Negative	0			
Positive	0.468	0.28		
Unknown	1.272	0.233		
**HER2**				
Negative	0			
Borderline	-1.038	0.206		
Positive	-0.671	0.141		
Unknown	0.825	0.392		
**Microscope**				
10X Microscope	0		0	
3.5X Loupes	-1.613	< 0.01	-1.516	< 0.01

Abbreviation: BMI, Body-mass index; HER2, human epidermal growth factor receptor 2

**Table 2B:** The uni- and multi- variate analysis of the ischemic time determinants

	Univariate		Multivariate	
	estimate	*p*. value	estimate	*p*. value
**Age**	1.285	0.03	1.353	0.01
**BMI**	-0.918	0.59		
**Neoadjuvant Chemotherapy**				
No	0			
Yes	-16.495	0.152		
Unknown	2.057	0.934		
**pT stage**				
pT0/T1/T2	0			
pT3/T4	-14.159	0.267		
pTx	-5.489	0.73		
**pN stage**				
N0	0		0	
N1-N3	27.69	0.02	16.697	0.12
Unknown	22.381	0.251	33.799	0.07
**Estrogen Receptor**				
Negative	0			
Positive	6.377	0.642		
Unknown	31.437	0.306		
**Progesterone Receptor**				
Negative	0			
Positive	22.576	0.05		
Unknown	41.021	0.206		
**HER2**				
Negative	0			
Borderline	-30.259	0.146		
Positive	-32.084	0.009		
Unknown	8.041	0.777		
**Microscope**				
10X Microscope	0		0	
3.5X Loupes	-52.755	0	-49.492	0

Abbreviation: BMI, Body-mass index; HER2, human epidermal growth factor receptor 2

### Exploratory analysis of the safety and feasibility of buried flap

After 80 cases, we incorporated the NSM into DIEP and buried the flap into the pocket without leaving any skin paddles, which were considered as a more challenging procedures for post-operative flap monitoring. The flap harvest time, ischemic time and surgery time of this approach showed a trend of decline in the learning curve (Fig. 1). After 4 cases of buried- flap breast reconstruction we reached the learning curve plateau soon. Within the buried-flap group, the median (IQR) surgery time and ischemic time were 7.0 (6.4–8.4) hours and 60.5(44.-74.3) minutes, respectively and both times were significantly shorter than the skin paddle group (Sup Fig. 1). The major and minor complications rates were similar between the two groups respectively. (Sup Fig. 2)

## Discussions

### Learning curve of MS-TRAM/DIEP surgery

MS-TRAM/DIEP is the mainstay approach for autologous tissue breast reconstruction with the advantages of maximizing the overall cosmetic outcomes of the reconstructed breasts. However, this approach has a learning curve that needs to be tackled. Our study showed that with accumulation of surgical experience after 30 cases, the surgery times and ischemic times were significantly reduced and were like previous studies [11–13]. The determinants of the surgery time were multi-factorial, such as unilateral or bilateral reconstruction, pre-operative CTA assessment or not, two microsurgeons operating at the same time with dedicated operative teams, etc. [14]. .

Complication rate was also a popular index to analyze the learning curve. Grinsell study reported that the complication rates were different between the first 30 flaps and the remaining 184 flaps, which was 10% vs. 7.6%, respectively [10]. Hofer’s study showed that the overall complication rate was different between the first 30 cases and the subsequent 144 cases, which was 40% vs. 13.8% [15]. Nieminen et al. demonstrated a 50% complication rate within the early 50 cases with eventual decrease to 20–25% [16]. In our study, the overall complication rate was similar between the first 30 cases and the remaining 117 flaps, which was 30.0% vs. 28.6%, suggesting that we reached the learning curve plateau at a relatively early time.

Reaching the plateau of the learning curve with accumulated experiences of the MS-TRAM/DIEP operation was the foundation to perform the buried-DIEP flap breast reconstruction with NSM. In our center, we performed the buried-DIEP surgery after 80 cumulative cases of experience, and we reached the buried-DIEP learning curve plateau after 4 cases. Within the buried-flap cohort, the senior dual-trained breast surgeons (Dr.LSR and Dr. ZLL) performed the DIEP only (No MS-TRAM) surgery by using 3.5X loupes exclusively. Using the 3.5X loupes and the younger age of patients were significant variable to the shorter operating time. DIEP is considered a harder surgical procedure than the MS-TRAM because of which needs more dedicate technique to dissect the pedicles. At the very early stage of our learning curve, we just did few cases of the MS-TRAM and then promote to DIEP surgery only at a relative short time. According to our learning curve, the DIEP dissection would not prolong the surgery time as our experience piling up. Meanwhile, the NSM preserves the original skin and contour of the breast borders, therefore saving overall surgery time that would otherwise be needed for breast reshaping. Haddock et al. mentioned that the operative setup and pre-operative decision-making would help to improve the efficiency of MS-TRAM/DIEP surgery to be less than 4 h [17].

### Pros and cons of leaving skin paddle and removing later vs. buried flap

The technique of buried flap versus flap with a monitoring skin paddle has sparked extensive discussions among researchers. In 2018, Frey et al. [7] conducted a comparative study on the safety of these two techniques and found that retaining the monitoring skin paddle offers better safety due to improved clinical observation and the possibility of secondary revision. Similarly, Park, in the field of head and neck surgery, highlighted the benefits of retaining the observation window for enhanced flap observation and salvage efficiency. However, the presence of a smaller skin paddle can impact the patient’s aesthetic outcome. In 2023, Hajime Matsumine’s team [18] discussed the use of a retained small skin paddle for nipple reconstruction, aiming to minimize scars, although some scarring is ultimately unavoidable. Nonetheless, retaining the skin observation window aligns with our clinical examination practices.

On the other hand, a totally buried flap offers the advantage of eliminating the need for secondary revision, and multiple studies have demonstrated its reliable safety, contributing to increased patient acceptance. In cases where a completely buried flap lacks a skin observation window, the use of implantable Doppler monitoring is recommended. However, it is important to note that research papers on implantable Doppler devices are predominantly published in Europe and North America [19]. In developing countries, the cost of such devices may be prohibitive, making them difficult to obtain. Consequently, in the absence of implantable Doppler devices, our focus in this article revolves around achieving comprehensive observation through intraoperative monitoring and traditional postoperative observation, drawing upon our collective experience.

The only take back case of the buried flap cohort was due to surgical field postoperative hemorrhage. The nurse noticed a bruise skin pocket with floating sign and a sudden increased blood drainage (over 200 ml / 60 min) after the 2 h of the patient returning to the ward. The patient was with an unstable vital sign, such as the increased heart rate (110 bpm) and low blood pressure(80/60mmHg). The Doppler signal was sounding more and more distant. We determine that was the postoperative hemorrhage and take back for emergency hemostasis. Within the surgery, we reperformed the strip test of the MS-TRAM/DIEP pedicles and then we made sure there was no thrombosis and venous congestion of the flap. The detailed intra- and post- operative protocol is listed within supplementary file (Supplementary File 1, Sup Fig. 3).

### Flap monitoring protocol in buried flap cohort

Buried flap breast reconstruction, though not increase the difficulty of surgical procedure, does increase the difficulty of post-operative monitoring. A traditional trans-cutaneous handheld doppler might not be sufficient to monitor the blood flow of the flap after anastomosis [20]. Vakharia reported the color doppler ultrasound was effective for the buried free flap moniroting [21] .Meanwhile, the implantable Doppler probes (Cook-Swartz) were invented for the specific purpose of the buried flap monitoring [22, 23]. However, a study by Whitaker et al. showed that implantable Doppler monitoring for patients with buried flap had significantly higher false positive rates and take-back surgery rates when compared with direct visual monitoring for patients with skin paddles [24]. In addition, the implantable Doppler might not be easily accessible in all hospitals, therefore, a validated protocol for buried flap monitoring without implantable Doppler was necessary. Levy reported that without implantable Doppler monitoring, buried flap was also feasible for breast reconstruction [25]. However, their study did not report a detailed protocol for intra-operative/post-operative flap monitoring. In our study, we proposed a validated protocol for MS-TRAM/DIEP flap buried into the NSM pocket, without implantable Doppler monitoring. We suggested that comprehensive, multi-dimensional intraoperative and post-operative monitoring would be effective. Furthermore, we noticed that several novel approaches could be incorporated to improve the efficacy and safety of our monitoring protocol. For example, indocyanine-green fluorescence video angiography, hydrogen clearance, CT angiography, MRI angiography, scintigraphy, micro-dialysis, and/or pH monitoring were all potential methods for blood flow monitoring [20]. Further studies are needed to address these methods.

### Incision of NSM with MS-TRAM/DIEP breast reconstruction

**Fig. 4:**
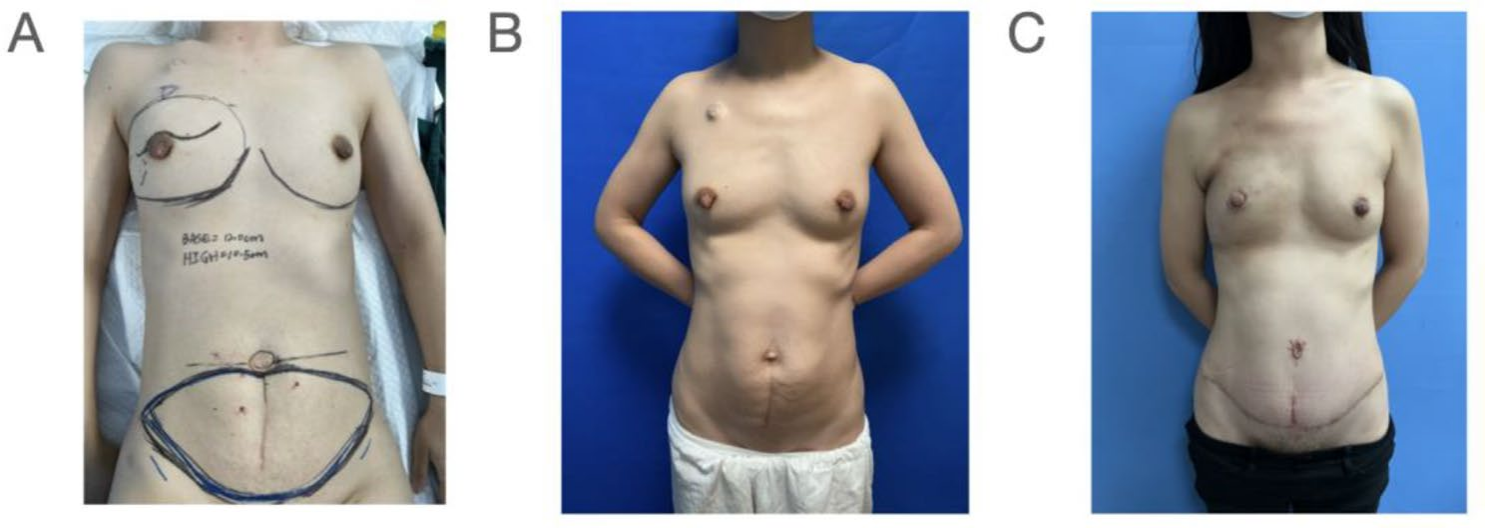
Skin marking of the Buried DIEP breast reconstruction. (**A**) The skin markings of the upper semi-peri areolar incision and the extension towards the 2nd and 3rd intercostal space (**B**) Preoperative picture (**C**) Six-month post-operative follow up

Selection of an appropriate incision for NSM is critical for buried-flap MS-TRAM/DIEP breast reconstruction. In our study, we used the peri-areolar incision as this incision is close to the internal thoracic vessels which facilitates the anastomoses (Fig. 4). However, when compared with IMF incision, peri-areolar incision might sacrifice part of the blood supply of the NAC. In our study, there were 38.9% (7/18) of NSM cases with epithelial and/or partial NAC necrosis, which led to compromised NAC shape or loss of pigmentation. There was no skin envelope necrosis or total nipple necrosis. Similarly, Levy et al. reported a 29.4% (5/17) risk of partial NAC necrosis in their study [25]. Thus, the balance between the “pros” (easy access to the internal thoracic vessels) and “cons” (increased risk of epithelial and/or partial NAC necrosis) of peri-areolar incision in NSM should be given focus. The superiority of the IMF in preserving the NAC blood supply can be noticed in the scenario of breast implant reconstruction. Colwell et al. reported that among the 285 patients with 500 NSM procedures, the rates of NAC necrosis that required surgical excision were 10.5% and 0.8% for peri-areolar incision and IMF incision, respectively [26]. The safety of the IMF incision was also demonstrated in a study reported by Yao. et al. in that 78.1% (310/397) of the NSM cases were performed via the IMF incision, leading to 1% (4/397) of NAC necrosis [27].

A disadvantage of IMF incision for NSM and MS-TRAM/DIEP breast reconstruction would be the difficulty of vessel anastomosis using the internal thoracic vessels as the recipient vessels. This might be overcome by robotic assisted surgery. Kuo et al. reported that robotic-assisted NSM through the anterior axillary line facilitated autologous flap breast reconstruction using the thoracodorsal (TD) vessels as the recipient vessels [28]. We proposed that NSM with the IMF incision might be possible for MS-TRAM/DIEP breast reconstruction using internal thoracic vessels as the recipient vessels with the help of robotic-assisted surgery in the future.

### Limitation

This article is a retrospective study with certain limitations. Firstly, our buried-flap cohort had a limited number of cases, and it requires a larger sample size to accumulate more clinical experience. Additionally, our patients did not complete follow-up using the Breast-Q questionnaire, making it difficult to determine which surgical approach, retaining the skin paddle for later excision or burying the flap without skin paddle, yields better aesthetic outcomes. Therefore, in the future, prospective comparative studies are needed to accurately assess whether the buried-flap technique leads to superior aesthetic results.

## Conclusion

This study sheds light on the learning curve experienced by dual-trained breast surgeons when performing MS-TRAM/DIEP breast reconstruction. The adoption of buried flap MS-TRAM/DIEP breast reconstruction was deemed safe once proficiency in the procedure was achieved.

### Electronic supplementary material

Below is the link to the electronic supplementary material.

Supplementary Material 1

Supplementary Material 2

Supplementary Material 3

## Data Availability

The datasets used and/or analysed during the current study available from the corresponding author on reasonable request.

## References

[1] Ouyang Q, Zhu L, Chen K, Su F (2015). Effect of implant vs. tissue reconstruction on cancer specific survival varies by axillary lymph node status in breast cancer patients. PLoS ONE.

[2] Huang NS, Wu J (2015). Nipple-sparing mastectomy in breast Cancer: from an oncologic safety perspective. Chin Med J (Engl).

[3] Mota BS, Riera R, Ricci MD, Barrett J, de Castria TB, Atallah AN, Bevilacqua JL (2016). Nipple- and areola-sparing mastectomy for the treatment of breast cancer. Cochrane Database Syst Rev.

[4] Galimberti V, Vicini E, Corso G, Morigi C, Fontana S, Sacchini V, Veronesi P (2017). Nipple-sparing and skin-sparing mastectomy: review of aims, oncological safety and contraindications. Breast.

[5] Fujimoto H, Ishikawa T, Satake T, Ko S, Shimizu D, Narui K, Yamada A, Sasaki T, Nagashima T, Endo I (2016). Donor site selection and clinical outcomes of nipple-areola skin-sparing mastectomy with immediate autologous free flap reconstruction: a single-institution experience. Eur J Surg Oncol.

[6] Stolier AJ, Sullivan SK, Dellacroce FJ (2008). Technical considerations in nipple-sparing mastectomy: 82 consecutive cases without necrosis. Ann Surg Oncol.

[7] Frey JD, Stranix JT, Chiodo MV, Alperovich M, Ahn CY, Allen RJ, Choi M, Karp NS, Levine JP (2018). Evolution in monitoring of free flap autologous breast Reconstruction after Nipple-Sparing Mastectomy: is there a best way?. Plast Reconstr Surg.

[8] Koshima I, Soeda S (1989). Inferior epigastric artery skin flaps without rectus abdominis muscle. Br J Plast Surg.

[9] Piper ML, Nathan S, Henderson S, Lee A, Broach RB, Kozak G, Davis H, Wu LC (2022). The impact of a single dual-trained surgeon in the management of Mastectomy and Reconstruction. Plast Reconstr Surg.

[10] Grinsell DG, McCoubrey GW, Finkemeyer JP (2016). The Deep Inferior Epigastric Perforator learning curve in the current era. Ann Plast Surg.

[11] Acosta R, Smit J, Audolfsson T, Darcy C, Enajat M, Kildal M, Liss A (2010). A clinical review of 9 years of free Perforator flap breast reconstructions: an analysis of 675 flaps and the influence of New techniques on clinical practice. J Reconstr Microsurg.

[12] Bodin F, Dissaux C, Lutz JC, Hendriks S, Fiquet C, Bruant-Rodier C (2015). The DIEP flap breast reconstruction: starting from scratch in a university hospital. Ann Chir Plast Esthet.

[13] Hultman CS, Kim S, Lee CN, Wu C, Dodge B, Hultman CE, Roach ST, Halvorson EG (2016). Implementation and analysis of a lean six Sigma Program in Microsurgery to Improve Operative Throughput in Perforator flap breast Reconstruction. Ann Plast Surg.

[14] Canizares O, Mayo J, Soto E, Allen RJ, Sadeghi A (2015). Optimizing efficiency in Deep Inferior Epigastric Perforator flap breast Reconstruction. Ann Plast Surg.

[15] Hofer SO, Damen TH, Mureau MA, Rakhorst HA, Roche NA (2007). A critical review of perioperative complications in 175 free deep inferior epigastric perforator flap breast reconstructions. Ann Plast Surg.

[16] Nieminen T, Asko-Seljavaara S, Suominen E, Kuokkanen H, von Smitten K (1999). Free microvascular tram flaps: report of 185 breast reconstructions. Scand J Plast Reconstr Surg Hand Surg.

[17] Haddock NT, Teotia SS (2021). Efficient DIEP flap: bilateral breast Reconstruction in Less Than four hours. Plast Reconstr Surg Glob Open.

[18] Matsumine H, Niimi Y, Jibiki N, Sakurai H (2023). Minimal scar autologous breast Reconstruction with skin-sparing mastectomy. Plast Reconstr Surg Glob Open.

[19] Chiesa-Estomba CM, Gonzalez-Garcia JA, Genden EM, Piazza C, Guntinas-Lichius O, Vander-Poorten V, Kowalski LP, Lopez F, Quer M, Rodrigo JP (2023). Complications related to the Cook-Swartz implantable doppler probe use in head and neck microvascular reconstruction: a systematic review. Eur Arch Otorhinolaryngol.

[20] Molitor M, Mestak O, Pink R, Foltan R, Sukop A, Lucchina S. The use of sentinel skin islands for monitoring buried and semi-buried micro-vascular flaps. Part I: Summary and brief description of monitoring methods. Biomed Pap Med Fac Univ Palacky Olomouc Czech Repub 2021. 10.5507/bp.2021.016 33821844

[21] Vakharia KT, Henstrom D, Lindsay R, Cunnane MB, Cheney M, Hadlock T (2012). Color Doppler ultrasound: effective monitoring of the buried free flap in facial reanimation. Otolaryngol Head Neck Surg.

[22] Parker PM, Fischer JC, Shaw WW (1984). Implantable pulsed doppler cuff for long-term monitoring of free flaps: a preliminary study. Microsurgery.

[23] Paprottka FJ, Klimas D, Krezdorn N, Schlarb D, Trevatt AEJ, Hebebrand D (2020). Cook-Swartz Doppler Probe Surveillance for Free flaps-defining pros and cons. Surg J.

[24] Whitaker IS, Rozen WM, Chubb D, Acosta R, Kiil BJ, Birke-Sorensen H, Grinsell D, Ashton MW (2010). Postoperative monitoring of free flaps in autologous breast reconstruction: a multicenter comparison of 398 flaps using clinical monitoring, microdialysis, and the implantable doppler probe. J Reconstr Microsurg.

[25] Levy J, Bosc R, Warren N, Rebecca S, Dao TH, Hersant B, Meningaud JP (2018). Nipple-sparing mastectomy and Immediate breast Reconstruction with a deep Inferior Epigastric Perforator flap: a study of patient satisfaction. Ann Plast Surg.

[26] Colwell AS, Tessler O, Lin AM, Liao E, Winograd J, Cetrulo CL, Tang R, Smith BL, Austen WG (2014). Breast reconstruction following nipple-sparing mastectomy: predictors of complications, reconstruction outcomes, and 5-year trends. Plast Reconstr Surg.

[27] Yao K, Liederbach E, Tang R, Lei L, Czechura T, Sisco M, Howard M, Hulick PJ, Weissman S, Winchester DJ (2015). Nipple-sparing mastectomy in BRCA1/2 mutation carriers: an interim analysis and review of the literature. Ann Surg Oncol.

[28] Kuo WL, Huang JJ, Huang YT, Chueh LF, Lee JT, Tsai HP, Chen SC (2020). Robot-assisted Mastectomy followed by Immediate Autologous Microsurgical Free Flap Reconstruction: techniques and feasibility in three different breast Cancer Surgical scenarios. Clin Breast Cancer.

