# Supplementary material for: The learning curve of the MS-TRAM/DIEP breast reconstruction by dual-trained breast surgeons
Source: BMC Surg. 2024 Feb 14;24:53. doi: 10.1186/s12893-024-02344-z (PMC10865591; doi:10.1186/s12893-024-02344-z)
Supplement: Supplementary file 1 — Supplementary Material 1 [file 12893_2024_2344_MOESM1_ESM.docx]

Supplementary Table

| Supplementary Table 1**.** The Learning Curve of the Buried DIEP *vs.* DIEP with Skin Paddle | | | |
| --- | --- | --- | --- |
|  | Buried flap cohort | Skin Paddle cohort | P-value |
| Surgery time,  median (IQR) hours | 7.0 (6.4-8.4) | 8.7(7.6-10.4) | <0.01 |
| Ischemic time,  median (IQR) minutes | 60.5(44-74.3) | 84(64.5-120.5) | <0.01 |
| *IQR：inter-quartile range | | | |
