# Supplementary material for: The learning curve of the MS-TRAM/DIEP breast reconstruction by dual-trained breast surgeons
Source: BMC Surg. 2024 Feb 14;24:53. doi: 10.1186/s12893-024-02344-z (PMC10865591; doi:10.1186/s12893-024-02344-z)
Supplement: Supplementary file 2 — Supplementary Material 2 [file 12893_2024_2344_MOESM2_ESM.docx]

Supplementary File 1

1. ***Preoperative surgical planning***

Preoperative abdominal CTA (at the thickness of 0.625-1.25 mm) was recommended for assessment of internal mammary vessels, as well as the trunk of the deep inferior epigastric vessels to determine the candidate donor/recipient vessels. The other potential donor and/or recipient vessels, e.g., superficial inferior epigastric vessels, intercostal vessels, and lateral thoracic vessels should also be checked. The optimal perforators on each side should be preoperatively marked on the skin (Figure 1A).

Surgical Procedures.

Two surgeons operated simultaneously, one performed the NSM, and the other surgeon harvested the DIEP flap at the same time. For NSM, we used the upper semi-peri areolar (upper side) incision. If the incision was not appropriate to expose the 2^nd^ and 3^rd^ intercostal space for anastomosis, we could extend the incision towards the upper inner direction (Figure 1A). The mastectomy was performed along the space between the Camper’s layer and Scarpa’s layer. We used Force FX electrosurgical device (SOMA TECH INTL) to dissect the skin envelope. Surgeons should pay attention to avoid thermal injury to the subdermal plexus(1). We performed sharp dissection of the NAC with scissors, and the retro-NAC tissues were collected and sent for frozen section analysis. The inframammary fold of the breast should be preserved during NSM.

For ms-TRAM/DIEP flap harvest, the pedicle of the deep inferior epigastric vessels should be dissected as long as possible. The surgery type of ms-TRAM or DIEP was determined based on the surgeons’ judgement. For anastomosis, we performed rib sparing techniques if possible. Microvascular anastomotic coupler was used for vein anastomoses under the 3.5X loupe magnification or 10X operating microscope based on surgeons’ preference.

1. ***Protocol of the intraoperative and post-operative flap monitoring***

2.1 Intraoperative monitoring

- Throughout the entire procedure: Mean arterial pressure should be maintained above 110 mmHg. Norepinephrine was not a contraindication, if required(2).
- Immediately after venous anastomosis, vascular patency test (Strip test, Sup Fig 1A) should be performed to ensure successful venous anastomosis(3, 4).
- 5-10 minutes after the anastomosis: Flap skin color assessment and capillary refilling test should be conducted to assess the efficacy of the vessels(5, 6).
- Before wound closure: De-epithelialization of the skin provides an opportunity to observe the efficacy of the vessel system after anastomoses. A “sweating-like” bleeding during de-epithelialization (Sup Fig 1B) indicates excellent blood flow and normal hydrodynamic pressure. If the bleeding is heavier or lighter than expected, re-exploration should be considered.
- After wound closure: A skin marking of the perforator site should be performed for post-operative sonograph monitoring.

2.2 Post-operative monitoring

We monitored the following checklist hourly for the 1^st^ day, and every 2-3 hours for the 2^nd^-3^rd^ post-operative day:

I) Symptoms: patients’ feeling of increased turgor pressure, pain, or change of the skin elasticity.

II) Vital signs: Accelerated heart rate (~110 bpm) and/or decreasing blood pressure indicates potential active bleeding. Hypotension after surgery may reduce flap perfusion and increase the risk of flap failure.

III) Drainage: Bloody drainage and/or sudden increased of drainage amount (~100 mL/h and/or non-stop dripping at 1 drip per second) indicates active bleeding or venous congestion.

IV) Breast skin pocket assessment: A bruised skin pocket with swollen breast indicates possible active bleeding and/or venous congestion.

V) Trans-cutaneous handheld doppler was used to monitor the vascular signals from the pedicle.

VI) Direct visual monitoring of the skin color (Not available for patients without skin paddles, Sup Fig 1C).

- Colored doppler ultrasonography was employed to check the bidirectional blood flow of the flap at 48 and 72 hours after surgery.
- 1. Abu-Hijleh MF, Roshier AL, Al-Shboul Q, Dharap AS, Harris PF. The membranous layer of superficial fascia: evidence for its widespread distribution in the body. Surg Radiol Anat. 2006;28(6):606-19.
- 2. Szabo Eltorai A, Huang CC, Lu JT, Ogura A, Caterson SA, Orgill DP. Selective Intraoperative Vasopressor Use Is Not Associated with Increased Risk of DIEP Flap Complications. Plast Reconstr Surg. 2017;140(1):70e-7e.
- 3. Ragnarsson R, Berggren A, Ostrup LT, Franzen L. Microvascular anastomosis of interpositional vein grafts with the UNILINK system. A comparative experimental study. Scand J Plast Reconstr Surg Hand Surg. 1989;23(1):23-8.
- 4. Krag C, Holck S. The value of the patency test in microvascular anastomosis: correlation between observed patency and size of intraluminal thrombus: an experimental study in rats. Br J Plast Surg. 1981;34(1):64-6.
- 5. Pickard A, Karlen W, Ansermino JM. Capillary refill time: is it still a useful clinical sign? Anesth Analg. 2011;113(1):120-3.
- 6. Sandberg LJ, Tzarnas, C.D. Assessment and interpretation of capillary refill in clinical evaluation of flap perfusion are subject to a lag time. European Journal of Plastic Surgery volume 2012;35(35):585-8.
