# Supplementary material for: The learning curve of the MS-TRAM/DIEP breast reconstruction by dual-trained breast surgeons
Source: BMC Surg. 2024 Feb 14;24:53. doi: 10.1186/s12893-024-02344-z (PMC10865591; doi:10.1186/s12893-024-02344-z)

Sup Fig.1 The surgery time (A)and the ischemic time(B) of the Buried Flap and the Skin paddle Flap group

A B

Sup Fig.2 (A) Major complications including the take-back rates and the flap failure rates were similar between the two cohorts. (B) Minor complications including the flap complications (*e.g.* liposclerosis, and flap volume decrease), the breast skin envelope complications (*e.g.* skin pocket necrosis) and the abdominal complication (*e.g.* complications of the abdominal incision and/or incisional hernia.


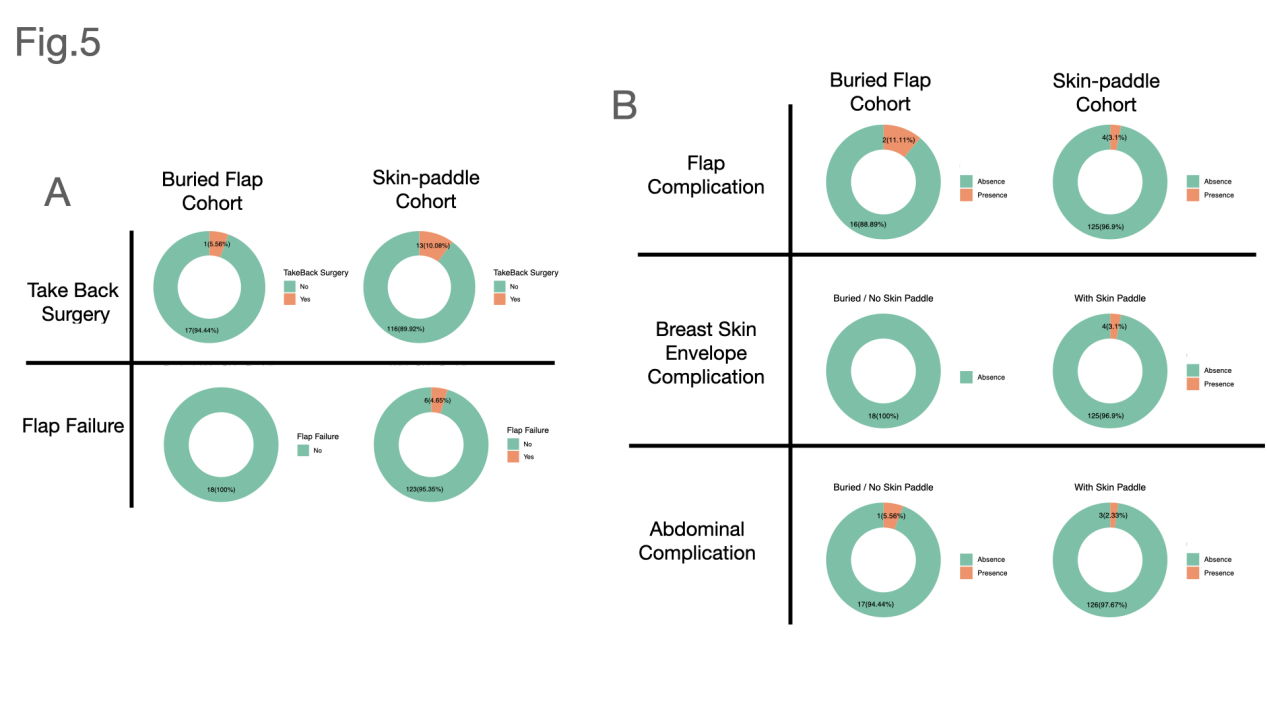


Sup Fig.3 Protocol of intra-operative and post-operative monitoring of the flaps. During anastomosis, the strip test (vascular patency test) was performed (LEFT). Before wound closure, the patterns of the bleeding during the de-epithelialization indicate different status of the blood flow (CENTER). After wound closure, the skin color assessment should be performed for post-operative monitoring (RIGHT).


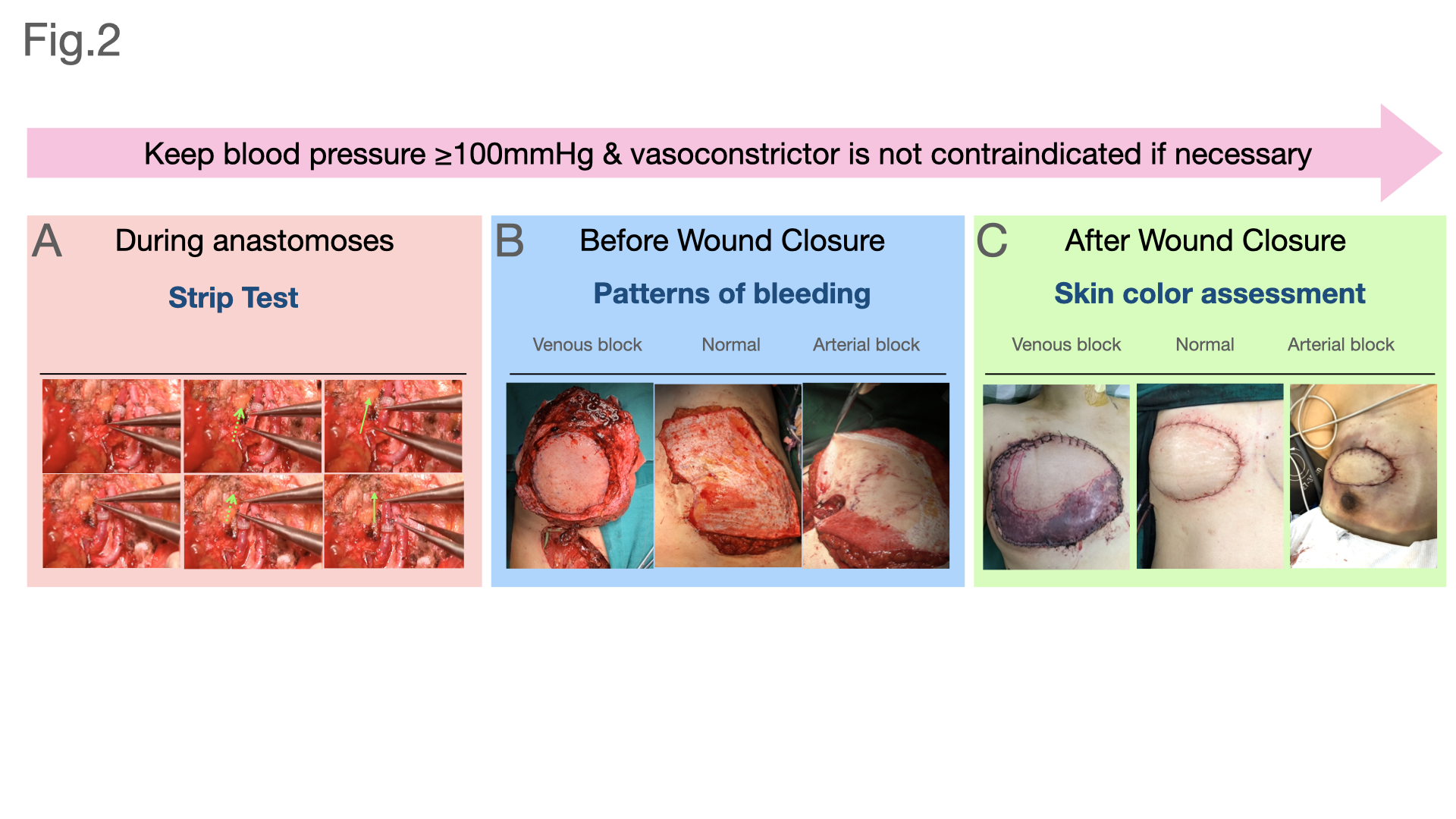

Supplement: Supplementary file 3 — Supplementary Material 3 [file 12893_2024_2344_MOESM3_ESM.docx]
